# 9/11-Related Experiences and Tasks of Landfill and Barge Workers: Qualitative Analysis from the World Trade Center Health Registry

**DOI:** 10.1186/1471-2458-11-321

**Published:** 2011-05-16

**Authors:** Christine C Ekenga, Katherine E Scheu, James E Cone, Steven D Stellman, Mark R Farfel

**Affiliations:** 1 Division of Epidemiology, New York City Department of Health and Mental Hygiene, New York, New York, USA; 2 Division of Epidemiology, Department of Environmental Medicine, New York University School of Medicine, New York, New York, USA; 3 Department of Environmental Health Sciences, School of Public Health, University at Albany, Albany, New York USA; 4 Department of Epidemiology, Mailman School of Public Health, Columbia University, New York, New York, USA

## Abstract

**Background:**

Few studies have documented the experiences of individuals who participated in the recovery and cleanup efforts at the World Trade Center Recovery Operation at Fresh Kills Landfill, on debris loading piers, and on transport barges after the September 11, 2001 terrorist attack.

**Methods:**

Semi-structured telephone interviews were conducted with a purposive sample of workers and volunteers from the World Trade Center Health Registry. Qualitative methods were used to analyze the narratives.

**Results:**

Twenty workers and volunteers were interviewed. They described the transport of debris to the Landfill via barges, the tasks and responsibilities associated with their post-9/11 work at the Landfill, and their reflections on their post-9/11 experiences. Tasks included sorting through debris, recovering human remains, searching for evidence from the terrorist attacks, and providing food and counseling services. Exposures mentioned included dust, fumes, and odors. Eight years after the World Trade Center disaster, workers expressed frustration about poor risk communication during recovery and cleanup work. Though proud of their contributions in the months after 9/11, some participants were concerned about long-term health outcomes.

**Conclusions:**

This qualitative study provided unique insight into the experiences, exposures, and concerns of understudied groups of 9/11 recovery and cleanup workers. The findings are being used to inform the development of subsequent World Trade Center Health Registry exposure and health assessments.

## Background

The terrorist attacks in New York City on September 11, 2001 resulted in one of the largest disaster recovery and cleanup efforts in United States history, which involved an estimated 91,500 workers and volunteers [1]. Much attention has been focused on the first responders and the workers and volunteers who participated in rescue and recovery activities at the World Trade Center site (Ground Zero) in lower Manhattan [2-4] and experienced persistent new or worsening respiratory symptoms, pulmonary function abnormalities, and chronic mental health morbidity [4-11] after 9/11.

Among rescue and recovery workers at Ground Zero, the 3-year risk of newly diagnosed asthma after 9/11 was estimated at 3.6%, approximately 12 times higher than the incidence of adult-onset asthma in the general U.S. population [4]. Early arrival after 9/11 and long duration of work days at Ground Zero were significant predictors of newly diagnosed asthma [4,10], persistent respiratory symptoms[6-8,11], and PTSD symptoms [5,9]. PTSD symptom estimates among World Trade Center (WTC) disaster workers have ranged from 6.2% to 21.2% [3,5,9,10], with a greater risk for developing PTSD reported among workers in occupations less prepared for disaster response, such as volunteers, construction workers, and sanitation workers [5].

Compared to Ground Zero workers, less is known about the experiences and health outcomes of barge and landfill workers who transported and processed more than 1.8 million tons of debris removed from Ground Zero as part of the recovery effort and the post-disaster criminal investigation [12]. Between September 12, 2001 and July 31, 2002, debris was transported by trucks to loading piers and then by barges to the WTC Recovery Operation at Fresh Kills Landfill on Staten Island, NY (hereafter referred to as the Landfill). Sifting and sorting operations at the Landfill yielded 4,257 human remains, 54,000 personal items, and pieces of material evidence from the terrorist attack [12].

Working conditions and the health outcomes of Landfill and barge workers may differ from those of Ground Zero workers. Exposures at Fresh Kills, including bacteria, diesel exhaust, dusts, heavy metals, and medical waste, have been a source of concern among Landfill workers even prior to 9/11 [13]. This qualitative study was designed to investigate the experiences and perceptions of persons enrolled in the World Trade Center Health Registry who performed 9/11-related work at the Landfill, on a debris loading pier or on a transport barge. This study was a crucial step in the development of a more in-depth job-exposure assessment for Landfill and barge workers.

## Methods

### Study Sample

The World Trade Center Health Registry (Registry) was established to follow the long-term health consequences of persons exposed to the September 11, 2001 terrorist attacks. The Registry includes a cohort of 71,437 individuals who enrolled and completed a health survey between September 2003 and November 2004, including 30,665 rescue, recovery, and cleanup workers and volunteers, of whom 4,490 (14.6%) reported working at the Landfill, on a loading pier in lower Manhattan, or on a barge operating between lower Manhattan and the Landfill [14]. Of these, a total of 911 (20%) workers and volunteers worked exclusively on a barge or at the Landfill.

Purposive sampling was undertaken to maximize the variety of perspectives. Participants were sampled by work site (Landfill, lower Manhattan loading pier, barge), organizational affiliation (Fire/Emergency Medical Services (EMS), Police, Sanitation, construction, public agencies, and volunteers), first date at work site (9/11/01, 9/12/01, 9/13/01 - 9/17/01, 9/18/01 - 12/31/01, and 1/1/02 - 6/30/02), and duration of work (days worked: < 7, 7-< 30, 30-< 90, and 90+).

Recruitment letters explained the purpose of the study and invited those interested to contact study investigators. Twenty recovery and cleanup workers and volunteers participated in the study out of a total of 44 recruited (response rate = 45.5%). A total of seven workers and volunteers declined to participate in the study, and the remaining 17 did not respond to recruitment efforts. All but two participants were men.

Participants included site supervisors, sanitation workers, police officers, and volunteers with broad experiences ranging from working a single day at the Landfill to working more than 90 days on a barge (Table 1). Some also reported participating in recovery and cleanup activities at Ground Zero such as digging through the rubble, providing peer counseling, serving food to the workers and volunteers, and looking for human remains in buildings in the vicinity of Ground Zero in lower Manhattan and beyond.

**Table 1:** Characteristics of the Study Population

Study Participant	Organizational Affiliation	Work Site(s)	Job Title	Work Duration
1	Construction	Barge	Rotating Foreman	>90 days

2	Construction	Landfill	Dump Truck Owner	>90 days

3	Fire Department	Landfill	Firefighter	>90 days

4	Fire Department	Landfill	Firefighter	7 - <30 days

5	Police Department	Landfill	Lieutenant	30 - 90 days

6	Police Department	Landfill	Police Officer	7 - <30 days

7	Police Department	Landfill	Police Officer	30 - 90 days

8	Police Department	lower Manhattan Pier	Sergeant	30 - 90 days

9	Public Agency	Landfill	Project Manager	>90 days

10	Public Agency	Landfill	Detective	7 - <30 days

11	Public Agency	lower Manhattan Pier	Director of Operations	>90 days

12	Sanitation	Barge and Landfill	Truck Driver	30 - 90 days

13	Sanitation	Barge and Landfill	Sanitation Worker	7 - <30 days

14	Sanitation	Landfill	Superintendent	30 - 90 days

15	Sanitation	Landfill	Supervisor	7 - <30 days

16	Sanitation	Landfill	Auto Mechanic	>90 days

17	Sanitation	lower Manhattan Pier	Sanitation Worker	30 - 90 days

18	Volunteer	Landfill	Truck Dispatcher	30 - 90 days

19	Volunteer	Landfill	Cook	<7 days

20	Volunteer	lower Manhattan Pier	Volunteer	<7 days

Informed consent was obtained from all study participants. Participants were interviewed by phone and sent a $10-$11 transit or gift card to thank them for their time. This study was approved by the Institutional Review Board of New York City Department of Health and Mental Hygiene.

### Data Collection

Semi-structured telephone interviews were conducted between May 2009 and July 2009 by two of the authors (CE and KS). An interview guide was developed to address study participants' involvement in and perspectives of disaster-related work away from Ground Zero after September 11, 2001. The interview guide was designed based on a review of the literature and feedback from labor and community advisors. The interview guide included a series of topics about 9/11-related work experiences: Employment Information, The Worksite, Daily Activities, Exposures, Protective Measures, and Concerns. Open-ended questions were used to elicit emerging topics of interest and allow participants to communicate their individual perspectives. Interview lengths ranged between 20 and 48 minutes. Interviews were audio recorded and transcribed verbatim for analysis.

### Data Analysis

Thematic analysis was used to examine interview content. Thematic analysis is a qualitative method for identifying, analyzing, and reporting patterns (themes) within a data set [15]. In this analysis, patterns across the different interviews were identified, and an "essentialist" approach was used to theorize experiences and perceptions [15]. The qualitative data analysis software, ATLAS.ti version 6.0 (Scientific Software Development Gmbh, Berlin, Germany), was used to analyze interview content.

Two coders (CE and KS) independently reviewed and coded each transcript. During the initial coding phase, a deductive approach was employed with the interview guide serving as the theoretical foundation from which principle thematic categories were identified. During the second coding phase, transcripts were further examined using an inductive analytical approach to identify additional themes. This phase of the analysis was data driven as themes identified during an inductive approach "bear little relation to the specific questions that were asked of the participants" [15]. Together, after this second review for emerging codes, both coders reviewed all codes, resolved any coding discrepancies, and refined the final coding frame. After final analyses of the narratives, a total of 27 codes (e.g., 'Flow of Debris', 'Job Tasks', 'Personal Protective Equipment', and 'Health Outcomes') were identified. Investigators then organized the 27 codes into common thematic categories.

## Results

Thematic analysis yielded nine common thematic categories. The themes were: Work at Ground Zero in lower Manhattan, Transport of Debris, Landfill, Tasks and Responsibilities, Reported Exposures, Protective Measures, Mask and Respirator Use, Participant Concerns, and Reflections on 9/11. Interrater reliability for each theme ranged from Kappa = 0.64 to Kappa = 0.97. Several themes were not among the topics included the interview guide. Reflections on the September 11 attacks, for example, was an example of a theme that was identified during the inductive coding phase. All nine themes are presented below, with representative quotations selected to highlight key results.

### Transport of Debris

**Figure 1:**
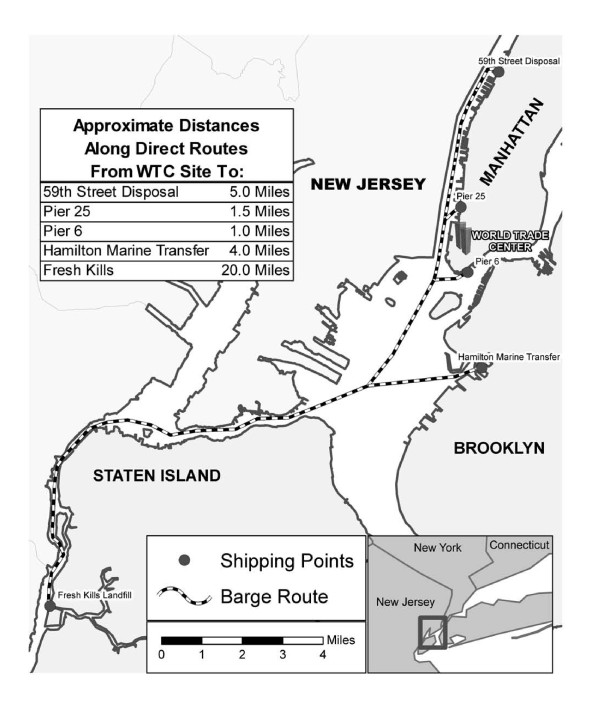
**Debris transport routes from World Trade Center site to World Trade Center Recovery Operation at Fresh Kills Landfill**.

Debris from Ground Zero was transported to lower Manhattan piers for loading onto barges (Figure 1). According to one pier worker: "they fill them [barges] up in Manhattan and then they bring them over to Staten Island." After the barges arrived on Staten Island, the barges were off-loaded by crane into a holding pit. One barge worker commented on the unloading process: "They [officials] would at different times be overseeing the unloading of a barge 'cause they thought things might be in it ...but they were just watching to see if there was anything of importance to them, that they would want this operation stopped and get everybody out of there and then they would be digging around for whatever it was they were trying to find." After the contents of the pit were inspected, the debris was loaded on trucks and driven to the Landfill.

### Landfill

Multiple participants commented on how disorganized the Landfill operation was in the first couple of weeks after September 11, 2001: "In the beginning it was chaos 'cause nobody really knew what was...they were doing the best they could to try to get everything organized and set up." A Landfill worker commented on the debris piles at the Landfill: "I mean like the first time I was there I mean the mounds of debris had to be...I don't know, it could be like three or four stories high above you and they would shake out all the debris - they would have these machines come and shake out debris and then we would walk through the debris..." Eventually, remarked another Landfill worker, the Landfill became "a small city within itself." Said a volunteer at the Landfill: "It looked like about three football fields long and they had a big tent up with detectives all dressed in their white uniforms, going through all the debris and whatnot, finding evidence-whether it be body parts or rings or wallets or whatever."

### Tasks and Responsibilities

Study participants were involved in a variety of activities at the Landfill. Reported tasks among Landfill workers included "driving tractor trailers", "fueling equipment", "burning steel", and "picking up plane parts for the FBI." Similar to Ground Zero work, some of the tasks included searching for human remains and evidence: "We sifted dirt to look through bones. Dug it up to put in pails, then you put the pails on the conveyer belt and you shifted around to take out what you thought were bones, bone fragments or whatever." Reported one police officer: "We were told to look for anything that would be of interest such as personal items to loved ones such as jewelry and we were looking for bone fragments or plane parts or whatever it might be, something that would help." One Landfill worker described sorting through the debris with rakes: "They had white buckets and you would put it in the bucket and you would bring it over and you would hand it in to the morgue area and hopefully they found out if it was somebody that they could identify."

Volunteers provided support services to the workers: "Red Cross was setting up food, massages, 'cause we stood on average the first month, for the first two months, you might have worked a 20-22- hour day. So they gave out food, coffee, sandwiches, water, massages, sometimes aspirin 'cause of course you have headaches because of no sleep." A peer counselor commented on his responsibilities: "[I] did some peer counseling in which we spoke to anybody that would listen - policemen, firemen, EMTs, civilians, construction workers, whoever wanted to listen we spoke to."

Most participants commented on the coordination between the different agencies and organizations operating at the Landfill. Remarked one firefighter: "We were all sorting and working together. Yes, I remember seeing police, construction workers and sanitation workers." Said a public agency official: "There was the fire, different police precincts from all over the country, fire department, emergency service, environmental protection, so many different agencies you can't list them all."

### Reported Exposures

All participants had comments about the dust and odors present during their recovery and cleanup efforts. One sanitation-affiliated participant said that there was a dust storm every day. Another sanitation worker reported that "it was a lot of dust and when the wind kicked up it was like the desert-you see the tumble weeds with all the dust coming around-it was like that." The dust was described as powdery, snow-like, chalky, and fine. Many were unsure about the sources of dust exposure. One participant, who worked on a barge and at the Landfill, noted: "Everything that was at the World Trade Center ended up...on the barge and came onto Staten Island. Everything that was there - the dust...the plaster, sand particles, whatever it was. It got loaded and then it got unloaded and it got put up on top of the hill." Another barge and Landfill worker stated that "we had a little bit of dust when we dumped out from the dust down at Ground Zero...but most of that dust was just dirt from driving over up to the top of the pile." A Landfill worker, who sorted through debris, said, "Occasionally there was some dust from machinery moving around and stuff coming down the conveyer belt."

The odor at the Landfill was reported as "indescribable," a "sick smell," a "methane smell", "the smell of garbage", and "the smell of death". A Landfill worker remarked that the mask protected him from smelling the odors, but a worker who was stationed at a loading pier stated that the smell, which he thought was from chemicals mixed with a powder smell, penetrated his mask. Other exposures reported included smoke exhaust, "toxins" in the air, smoldering steel, gasoline, welding fumes, asbestos, and methane gas bubbling from the ground. One participant, who worked as a cook at the landfill for less than seven days, did not view air quality as a problem.

### Protective Measures

There were varying descriptions of the health and safety measures undertaken during recovery and cleanup. A majority of study participants reported that they wore protective suits at the Landfill (most mentioned wearing Tyvek suits and one reported wearing a Nomex suit), many reported wearing protective boots, several reported using gloves, and one participant stated that helmets and goggles were distributed. One Landfill worker, who volunteered as a peer counselor, mentioned not using any special uniforms or protective gear.

More than half of the participants who worked at the Landfill reported seeing or using wash stations that were set up for workers to use before they entered and exited the Landfill itself or before they entered Landfill mess halls. According to one police officer, "We went into a shed and I think we took off the gloves, took off the Tyvek suit, but left the boots on, and somebody washed the boots with some sort of bubbly type of cleaner in a bath, and then you went into a different foot bath where they washed that off, and then out into the area where we were eating." A sanitation worker noted that there were shower facilities on site: "It was part of our work site. We had lockers rooms, showers, bathrooms, the whole works." Though multiple participants reported using or seeing showers at the site, three reported that they did not use or see any showers.

Participants reported different approaches to discarding their work clothing off-site. Some did not mention any special measures, whereas others went to great lengths to separate their clothing from that of others: "My work clothes during that time was never washed in my house. She would wash it downstairs in the laundry. My work boots, I would take them off and leave them in the car ...But nothing from the actual 9/11...I didn't bring any of that into my house."

### Mask and Respirator Use

The majority of study participants reported using a mask during at least some part of their post-disaster work; two reported not being given a mask at all. One police officer was given a mask at Ground Zero, but not at the Landfill. Of the participants who stated that they used a mask or respirator, most were fit-tested and trained on their usage. Remarked one Landfill worker: "...they had people from I think OSHA there and they would fit. It was like maybe I would say maybe a month or so after the original thing started. You'd walk over there and they would burn smoke in front of you and they would have you have the mask on. If you had smoke coming in, breathing it, they would adjust the mask accordingly." Half of those who mentioned using masks remarked that they used "paper dust masks" for a period of time before they were given respirators: "...in the beginning, we were just given the little dust mask, like you would wear if you were in a shop or mowing your lawn or something, and... that lasted for a long time before they started to get these I don't even know what...they're called...anyway, advanced masks." Four of the Landfill workers noted that they received their masks the first day of work at the site.

According to study participants, enforcement of mask and respirator use varied: a Landfill worker in a supervisory position was told a mask was not needed for his job: "I requested a mask but they had me speak into a walkie-talkie all day. They told me it wasn't required that I had one. Even though I requested one, I never got one." Two participants stated that OSHA or site supervisors could enforce mask use. According to one supervisor, "we were also told - oh yeah, you got to make sure everybody wears their masks and suits and if they don't, you can do whatever you got to do to them, you know give them complaints or send them off the job or whatever it was." A couple of participants reported that they did not wear any masks or respirators because of their jobs and responsibilities: "the problem was, for me, was that I was on the radio all the time 'cause I was foreman...for me I'd always have to pull the mask down and talk on the radio, so it was very difficult to...communicate with that on my face."

### Participant Concerns

A source of frustration mentioned by some participants was the lack of communication about potential health and safety hazards associated with their recovery and cleanup responsibilities. A Landfill worker commented on inconsistent information: "there were a lot of passenger vehicles that were brought out of the World Trade Center and brought over there [to the Landfill]. And in the beginning everybody thought there was[n't] anything wrong with this, just rinse them off and let these people have their cars back...Well later on they said, oh not a good idea - there could be all these contaminants in there, but again, in the beginning nobody realized this. But we were all told, it wasn't there. There was no contamination here, and then later on, it got to be a big thing." Another Landfill worker described his outrage at public officials: "I just don't understand anything why, again, nobody knew anything for a month and a half, we were all told that there's nothing wrong here. This is just a dust problem, you know, protect your eyes and wear a dust mask...there's nothing really bad here. And that's not quite the case. And I never saw it followed up or anybody go back and say, well why did you say this, or who told you to say this?"

Participants commented on their current physical health and mental well-being. Most reported that they were in good health. Some reported post-9/11 physician-diagnosed illnesses such as asthma, bronchitis, and sleep apnea. Two participants reported a post-traumatic stress disorder (PTSD) diagnosis after 9/11. Said one : "I've [got] post-traumatic stress disorder. I was diagnosed with that, major depression, all that kind of stuff, counseling, all. I actually filed for disability related to the PTSD."

Some participants expressed concerns about future health outcomes. One participant expressed concern about his health after participating in Landfill cleanup activities: "...the only thing that changed since that happened is I have shortness of breath and stuff like that but nothing I can't deal with it. What concerns me is later on in life. Like I said, it never bothered me up until I had a baby and now you start thinking about the future." Another commented on his need for counseling: "I'm also concerned about my mental well-being as time goes on as well. Even today, I don't know if I'm ready to talk to somebody about it, but I need to talk about this stuff in the future."

### Reflections on post-9/11 Experiences

The interviews reflected a tremendous amount of pride among the participants. A Landfill worker commented: "...For the most part, we got the job done and I guess what should have been probably the world's biggest hazmat job turned into a nine-month clean-up. It should have taken two years..." Many participants spoke about their recovery and cleanup experiences and the impact of their efforts. Said one volunteer: "...It was rewarding for me to be able to help the people who were kind of working down there. Being somebody who was there on September 11th, it kind of gave me a little bit of closure in being able to kind of help where I could." A barge worker commented on his colleagues: "I get a little choked up thinking about it, I'm sorry. But I met some wonderful people down there that just came down to help out and try to do whatever they could to make things better down there. A lot of people did, and they made a big difference." One Landfill worker who was present at Ground Zero on September 11^th ^reflected on the events of that day: "I probably saw the worst of humanity and then within a matter of 24 hours I saw the best of humanity."

## Discussion

This qualitative study complements studies of the experiences of Ground Zero workers [2,16] by elucidating the experiences of people who participated in recovery and cleanup activities away from Ground Zero, at the WTC Recovery Operation at the Fresh Kills Landfill, on debris loading piers, and on transport barges. Some participants noted that in the days immediately following the disaster, the Landfill was chaotic and disorganized. Others noted that it was not until weeks after the disaster that the worksite was considered organized and fully operational. Consistent with reports from Ground Zero workers [16], study participants expressed frustration about a lack of personal protective equipment (PPE) and training, yet all but two reported using a mask or respirator during their recovery work. Though responses varied regarding mask and respirator type, some participants reported that they had not been trained to use respirators, or if they did receive training, the tasks and responsibilities associated with their work made it difficult for them to wear their masks and respirators consistently.

Several participants were concerned about the long-term physical and emotional health consequences of their WTC-related work and the impact that their impaired health may have on their families and loved ones. In the New York City metropolitan area and nationally, the National Institute for Occupational Safety and Health (NIOSH) has provided funding for programs that provide medical monitoring and treatment to WTC responders[17]. In December 2010, the United States Congress established a long-term program to provide medical monitoring and treatment benefits to 9/11 emergency responders and recovery and cleanup workers, singling out those who participated at the Staten Island Landfill, or the barge loading piers as eligible beneficiaries. A section of this law sets an agenda for research on 9/11 related health conditions that may be eligible for treatment [18]. The Landfill and barge worker component of the Registry is the largest source of data available for that purpose. Nine years after the World Trade Center disaster, potential adverse health outcomes associated with Landfill and barge worksite exposures, particularly dust, fumes, and odors, remain a source of concern.

It is important to note that interviews of Landfill and barge workers revealed an immense sense of pride in their contributions to the WTC disaster recovery and cleanup efforts. Their work was intense and demanding and involved daily contact with large volumes of debris containing potential human remains and personal effects that continued for months after September 11, 2001. Study participants often expressed their respect and admiration for the courage displayed by their coworkers after the disaster.

The current study enabled us to gain new insight into the tasks, responsibilities, and types of exposures among landfill and barge workers after September 11, 2001. Results from this study have been used to facilitate a focused survey of all 4490 Landfill and barge workers in the WTC Health Registry, and study results were essential in developing detailed task and exposure questions. This included a checklist of 17 tasks and specific questions about exposures such as dust, smoke, metal fumes, gas fumes, and human remains. Based on participant comments, we plan to include questions about disaster response training and experience prior to September 11, 2001 to gauge the preparedness among this group of disaster workers. In addition, to address the diverse experiences with personal protective equipment, we have developed questions about PPE type, PPE availability, and the frequency and duration of PPE use.

Strengths of this qualitative study include the ability to capture the unique reactions to the WTC disaster from the perspectives of this small sample of landfill and barge workers, an under-examined population. Limitations of this study should be noted. Workers and volunteers were self-selected, and interviews were conducted 7-8 years after their WTC experiences. Their anecdotal accounts of their experiences may have been compromised by the length of time between post-disaster work and the telephone interview. These interviews, however, were able to capture a wide variety of work experiences through a diverse group of workers and volunteers enrolled in the Registry. Stratified sampling by worksite and organizational affiliation, in particular, was useful in gathering data on a wide range of tasks and activities. As a result, we were able to investigate past tasks and responsibilities, types of exposures, and current concerns of landfill and barge workers.

## Conclusions

The qualitative analysis of the narratives of landfill and barge workers allowed for a fuller understanding of the tasks and experiences of an understudied subgroup of World Trade Center recovery and cleanup workers and volunteers. The perspectives of landfill and barge workers have been largely missing from World Trade Center studies. The Registry has used these first-person accounts to develop an exposure questionnaire that will further clarify our understanding of the complex multi-step cleanup and recovery work conducted by landfill and barge workers for nine months in the wake of 9/11 in a way that will inform subsequent risk assessments in this population. Our findings may also provide guidance for designing evaluations of future disaster response efforts.

Our research highlights the utility of qualitative analyses in exposure assessments after a disaster. Combined with epidemiologic studies, these narratives will contribute to a better understanding of reported exposures and health outcomes among 9/11 recovery and cleanup workers. Further studies are underway to examine the relationship between post-disaster exposures and health outcomes in this population.

## Competing interests

The authors declare that they have no competing interests.

## Authors' contributions

CE participated in the design of the study, data collection, data analyses, drafted the original manuscript, and critically reviewed and revised the manuscript. KS participated in data collection, data analyses, drafted the original manuscript, and critically reviewed and revised the manuscript. JC obtained funding, participated in the design of the study, and critically reviewed and revised the manuscript. SS obtained funding, participated in the design of the study, and critically reviewed and revised the manuscript. MF obtained funding, participated in the design of the study, and critically reviewed and revised the manuscript. All authors have read and approved the final manuscript.

## Pre-publication history

The pre-publication history for this paper can be accessed here:

http://www.biomedcentral.com/1471-2458/11/321/prepub
